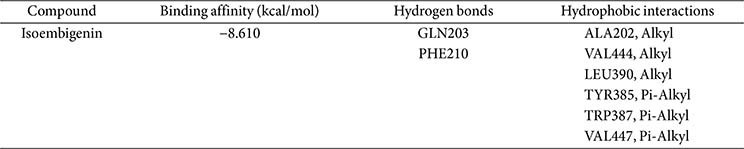# Exploring the Anti-Inflammatory Potential of Isoembigenin: A Flavonoid Glycoside from *Piper aduncum* L. – A Case Exploration through *In Vitro*, *In Vivo*, and In Silico Models

**DOI:** 10.4014/jmb.2505.05023

**Published:** 2025-10-15

**Authors:** Nguyen Thi Minh Nguyet, Vinh Le Ba, Van Dinh Nguyen, Cuong Nguyen Cao, Huong Dinh Thi, Phong Nguyen Viet, Luyen Bui Thi Thuy, Chang-kyu Lee, So Young Ban, Jong-Tae Park

**Affiliations:** 1 Vinmec-VinUni Institute of Immunology, College of Health Sciences, VinUniversity, Hanoi 100000, Vietnam; 2 CARBOEXPERT Inc., Daejeon, Republic of Korea; 3 University of Medicine and Pharmacy, Vietnam National University, Hanoi, Vietnam; 4 Faculty of Medicine and Pharmacy, Yersin University of Da Lat, Lam Dong, Vietnam; 5 Department of Biology Education, Teachers College and Institute for Phylogenomics and Evolution, Kyungpook National University, Daegu 41566, Republic of Korea; 6 Department of Pharmaceutical Industry, Hanoi University of Pharmacy, Hanoi 100000 Vietnam; 7 Department of Food Science and Technology, College of Agriculture, Chungnam National University, Republic of Korea; 8 Center of Allergy and Clinical Immunology, Vinmec International Hospital, Hanoi, Vietnam

**Keywords:** Isoembigenin, sepsis murine model, anti-inflammation, RAW 264.7, tumor necrosis factor alpha (TNF-α), interleukin-1beta (IL-1β)

## Abstract

*Piper aduncum* L. has long been used in traditional medicine for its notable antimicrobial, anti-diarrheal, and anti-inflammatory properties. Flavonoids are recognized as the principal bioactive constituents of this plant, among which isoembigenin (IEGN) has been shown to inhibit dendritic cell–mediated inflammatory responses. However, its effects on other immune cell types involved in inflammation remain unclear. In this study, we examined whether IEGN pre-treatment could attenuate macrophage activation triggered by lipopolysaccharide (LPS). Our results demonstrated that IEGN significantly suppressed TNF-α expression in RAW 264.7 macrophages upon LPS stimulation. Moreover, in a murine sepsis model, IEGN pre-treatment markedly reduced the production of proinflammatory cytokines, including TNF-α and IL-1β, shortly after LPS challenge. Importantly, IEGN administration substantially improved the survival rate of BALB/c mice subjected to endotoxin-induced shock. Finally, the molecular docking simulation was a hypothetical assessment of the interaction between IEGN and iNOS and COX-2. Taken together, these findings highlight the therapeutic potential of IEGN as a candidate for preventing inflammation driven by macrophage activation.

## Introduction

Inflammation plays an important role in the innate immune response. Sepsis is an exaggerated systemic inflammatory response that leads to profound consequences, such as multi-organ dysfunction, increased healthcare costs, and even a high mortality rate of up to 10% in hospitalized cases [1]. The pathogenesis of sepsis is highly multifaceted and involves several different mechanisms, such as infection, inflammation, immune cells including macrophages, blood coagulation, and endothelial dysfunction [2]. Among systemic inflammatory models, the endotoxemia model using lipopolysaccharide (LPS) as a component of the outer membrane of Gram-negative bacteria has already been established [3].

In the action mechanism of the endotoxemia model, after entering the body, macrophages are activated and subsequently differentiate into M1 macrophages due to LPS binding to toll-like receptor 4 (TLR-4) on the cell membrane. These TLR-4 receptors can recognize and bind to LPS, thereby activating three inflammatory signaling pathways in macrophages: NF-kB, MAPKs, and JAK/STAT, which lead to the production of pro-inflammatory cytokines such as TNF-α, IL-6, IL-1β, and monocyte chemotactic protein-1 (MCP-1) [4-6]. The accumulation of these inflammatory mediators results in increased tissue and cellular damage, which can promote a severe inflammatory response. Previous studies have shown that LPS mediates early direct injury to multiple organs by triggering the inflammatory cell system to produce cytokines that peak earlier, at higher concentrations, and for a shorter duration in the context of endotoxemia [7-10]. In addition, high doses of LPS cause hypotension and hypothermia, which are characteristic of murine septic shock.

Although there are well-established strategies aimed at treating the underlying infection, the development of new therapeutic options and the identification of potential drug candidates are urgently needed to prevent and combat sepsis. For this purpose, animal models can be very useful for conducting preclinical tests on purified compounds from traditional plants that have been identified as rich sources of natural products with anti-inflammatory activities.

*Piper aduncum* L. has been prevalent in Asian countries for thousands of years, serving both as a spice and as a traditional herbal medicine. In addition to its seeds, the leaves of *P. aduncum* are widely used in folk medicine. Recent scientific studies provide modern evidence supporting its antimicrobial, anti-diarrheal, and anti-inflammatory properties [11]. Isoembigenin (IEGN), a C-glycoside flavone isolated from the leaf extracts of *P. aduncum*, has previously demonstrated promising anti-inflammatory effects mediated by dendritic cells [12]. In inflammatory mechanisms, dendritic cells primarily activate T cells, while macrophages eliminate apoptotic cells and microbes through phagocytosis [13]. Against this background, our current investigation explored the impact of IEGN on macrophage cells within a murine sepsis model.

## Materials and Methods

### Plant Material

The leaves of *Piper aduncum* were collected from Coblong, Bandung, West Java, Indonesia, in August 2022 and were taxonomically identified by Dr. Le Ba Vinh. A voucher specimen (PA 08) has been deposited in the Herbarium of the Department of Food Science and Technology, College of Agriculture, Chungnam National University.

### Isolation of Active Compounds

**Fig. 1:**
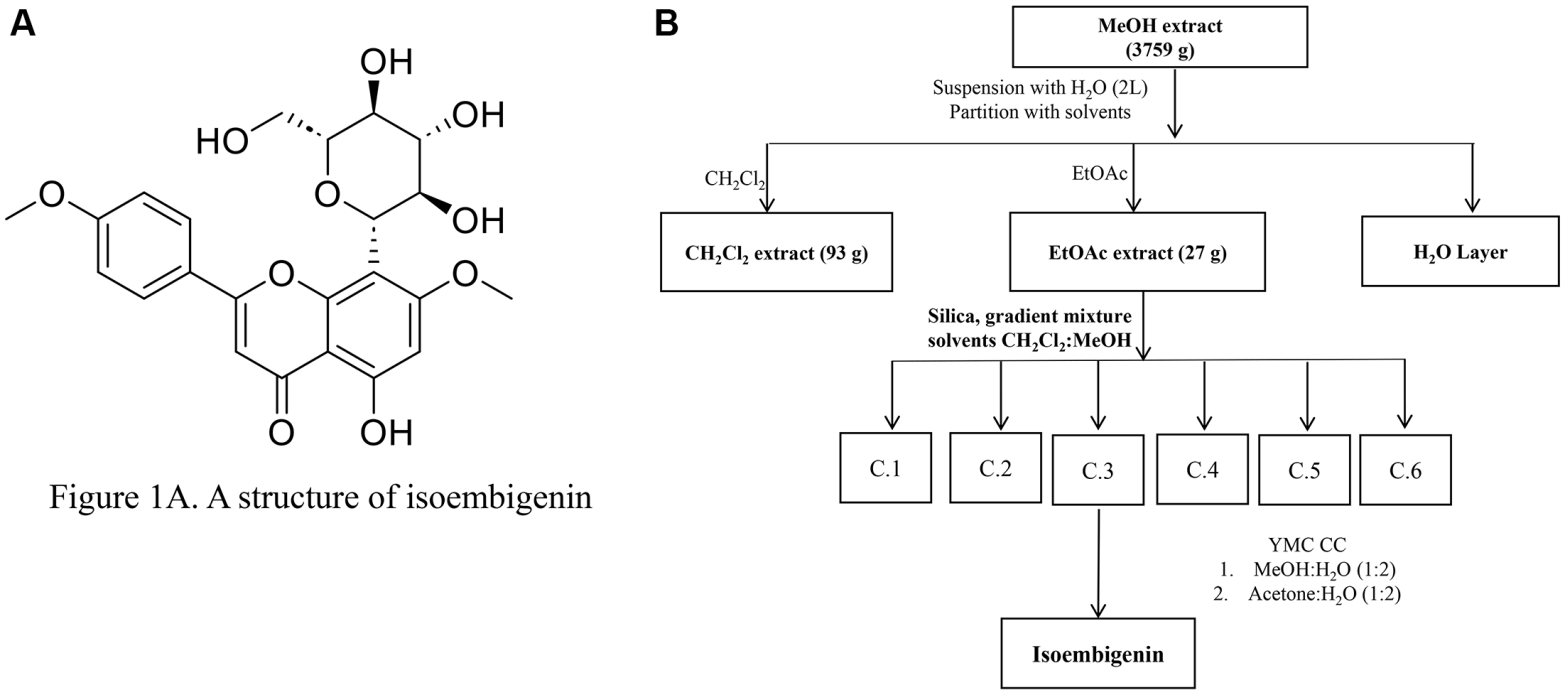
Scheme of isoembigenin isolation process. (**A**) Determination of the chemical structure of isoembigenin. (**B**) Extraction and isolation of isoembigenin from *Piper aduncum* leaves using combined column chromatography with the ethylacetate (EtOAc) fraction.

The dried leaves of *P. aduncum* (3.2 kg) were extracted with methanol (10 L × 3 times) under reflux conditions. Evaporation of the solvent under reduced pressure yielded a crude extract (379 g), which was suspended in H_2_O and successively separated with CH_2_Cl_2_ and Ethyl Acetate, resulting in CH_2_Cl_2_ (93 g) and EtOAc extracts (27 g). The EtOAc extract (27 g) was fractionated by silica gel column chromatography (CC), eluting with a gradient solvent system of CH_2_Cl_2_-MeOH (0–100% MeOH, stepwise) to obtain six fractions (C.1 through C.6). Isoembigenin (20 mg) was isolated from fraction C.3 using YMC reverse-phase (RP)-18 CC (YMC*GEL (ODS-A, 12 nm S-150 mm, YMC)) with MeOH-H_2_O (1/1, *v/v*) as the eluent and further purified by YMC RP-18 CC eluting with acetone-H_2_O (1/2, *v/v*) (Fig. 1).

### Cell Viability Assay

The safety of the compound on cell viability was determined using an MTT: 3-(4,5- Dimethylthiazol-2-yl)-2,5-Diphenyltetrazolium bromide assay. Murine macrophage cells (RAW 264.7) were seeded into a 96-well plate at a density of 1 × 10^5^ cells per well. Different concentrations of isoembigenin (IEGN) ranging from 3.125 μM to 100 μM were treated to the cells for 6 h. Subsequently, 10 μl of MTT stock (Sigma Aldrich, USA) (5 mg/ml) was added to each well and incubated for 2 h. After removing the supernatant, the formazan crystals were dissolved in 100 μl of Dimethyl sulfoxide, (Sigma Aldrich) at room temperature. The absorbance was measured with a microplate reader at 570 nm [14].

### Cytokine Measurement Methods

RAW 264.7 cells were seeded in a 96-well culture plate at a final concentration of 1 × 10^5^ cells per well. They were incubated for 1 h at 37°C, followed by pretreatment with IEGN (ranging from 3.125 μM to 100 μM) for 30 min and subsequent stimulation with LPS (Sigma Aldrich) at 1 μg/ml for 6 h. The level of TNF-α was measured in the culture supernatant using Legend Max Mouse TNF-alpha ELISA kit (Biolegend, USA) [15].

### *In Vivo* LPS-Induced Sepsis Murine Model

**Animal.** Male BALB/C mice (6 weeks of age) were purchased from SAMTAKO Company (Republic of Korea) and maintained at Chungnam Animal Center under pathogen-free conditions. All the mice were provided with sufficient food and water and were allowed to rest for 1 week. All animal experiments were conducted in compliance with a protocol approved by the Institutional Animal Care and Use Committee of Chungnam National University (202203A-CNU-056).

**LPS model of systemic sepsis.** Briefly, the 3 mice were divided into groups. IEGN was prepared using a Tween 20-saline solution (1:99) and administered orally to the mice for 4 consecutive days. Dexamethasone was dissolved in distilled water and also administered orally to the mice for 4 consecutive days. The naïve and sham groups received the equivalent vehicle solution instead. One hour after the last pretreatment, LPS was injected intraperitoneally(i.p.) into the mice, except for the naïve group, at a final concentration of 5 mg/kg. The sham group served as the positive control group. Blood samples were taken 2 h and 6 h after the LPS injection, and the concentrations of TNF-α and IL-1β were measured using ELISA kits (BioLegend, USA). Other clinical symptoms were monitored every 2 h over a 1-day period, including changes in rectal temperature, diarrhea, survival rate, and spleen inflammation. The rectal temperature was measured 4 h after LPS-induced inflammation using a rectal probe thermometer (Physitemp, USA). Diarrhea was assessed by visually monitoring the mice for up to 24 h after LPS injection. Mice exhibiting profuse liquid stools were recorded as having diarrhea. Diarrhea severity was scored as follows: 0, normal stools; 1, watery, unshaped stools; 2, watery and frequent stools; 3, death.

### Statistical Analysis Methods

Comparisons between the negative control group and treatment groups were made using nonparametric one-way ANOVA (Kruskal-Wallis test) and confirmed with Bonferroni correction as a post hoc analysis. A *p*-value of < 0.05 was considered statistically significant.

### Molecular Docking Techniques

Binding interactions between the isolated compounds and selected proteins were analyzed through molecular docking simulations using AutoDock Vina 1.1.2 (Molecular Graphics Laboratory, The Scripps Research Institute), following established methodologies from previous studies [16, 17]. The X-ray crystallographic structures of inflammation-related proteins, including iNOS (PDB ID: 4NOS) and COX-2 (PDB ID: 5F1A), were downloaded from the RCSB Protein Data Bank [18]. The three-dimensional structures of the test compounds were built using Chem 3D Pro version 2022 (PerkinElmer Informatics, Version 22.2) after energy minimization. The 2D (two-dimensional) and 3D docking images of the active compounds were created using Discovery Studio (v21.1.0.20298, Dassault Systemes Biovia Corp) and PyMOL software (Schrodinger, LLC. version 2.5.7 for educational use), respectively [19].

## Results

### *In Vitro* Inhibition of Proinflammatory Cytokines by Isoembigenin in Macrophage Cells

**Fig. 2:**
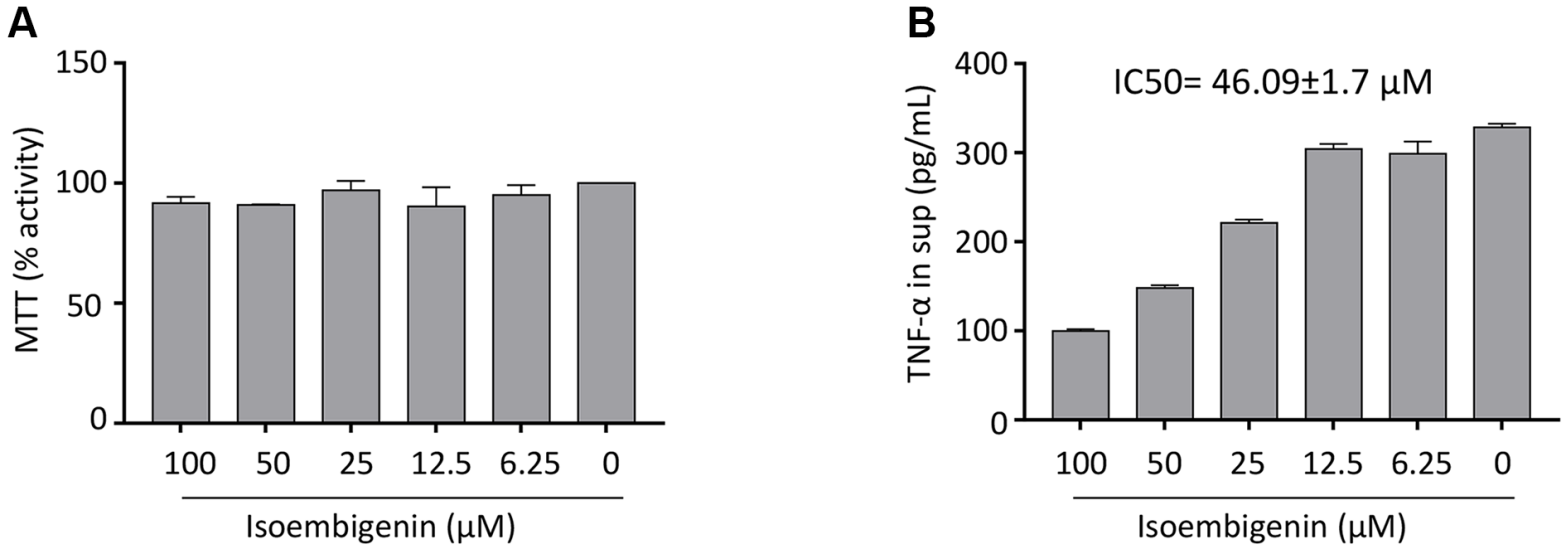
Effects of isoembigenin on the productions of TNF-α by LPS-activated murine macrophages cell line (RAW 264.7). Cells were incubated with different concentrations of compounds, and then were stimulated with LPS (1 μg/ml) for 6 h. The culture supernatant was harvested and stored at -80°C before ELISA. (**A**) The cytotoxicity of compounds was measured by MTT assay and (**B**) the level of TNF-α was measured by ELISA . The data are presented as the means ± SD of three independent experiments.

Before conducting further experiments, the cytotoxicity of IEGN on cells was assessed. RAW 264.7 cells were seeded into a 96-well plate and treated with different concentrations of the compound for 6 h. IEGN-induced cytotoxicity was measured using the MTT assay. The results showed that the survival rate of RAW 264.7 cells did not differ significantly with or without IEGN treatment (Fig. 2A).

TNF-α is a key pro-inflammatory cytokine released during macrophage activation. We investigated the inhibitory activities of IEGN on TNF-α secretion stimulated by the RAW 264.7 cell line. As shown in Fig. 2B, when RAW 264.7 cells were pre-treated for 1 hour with different concentrations of IEGN before LPS stimulation for 6 h, IEGN exhibited strong TNF-α inhibitory activity, with an IC_50_ value of 46.09 ± 1.7 μM.

### Reduction of Endotoxemia Mortality by Orally Administered IEGN in a Mouse Model

**Fig. 3:**
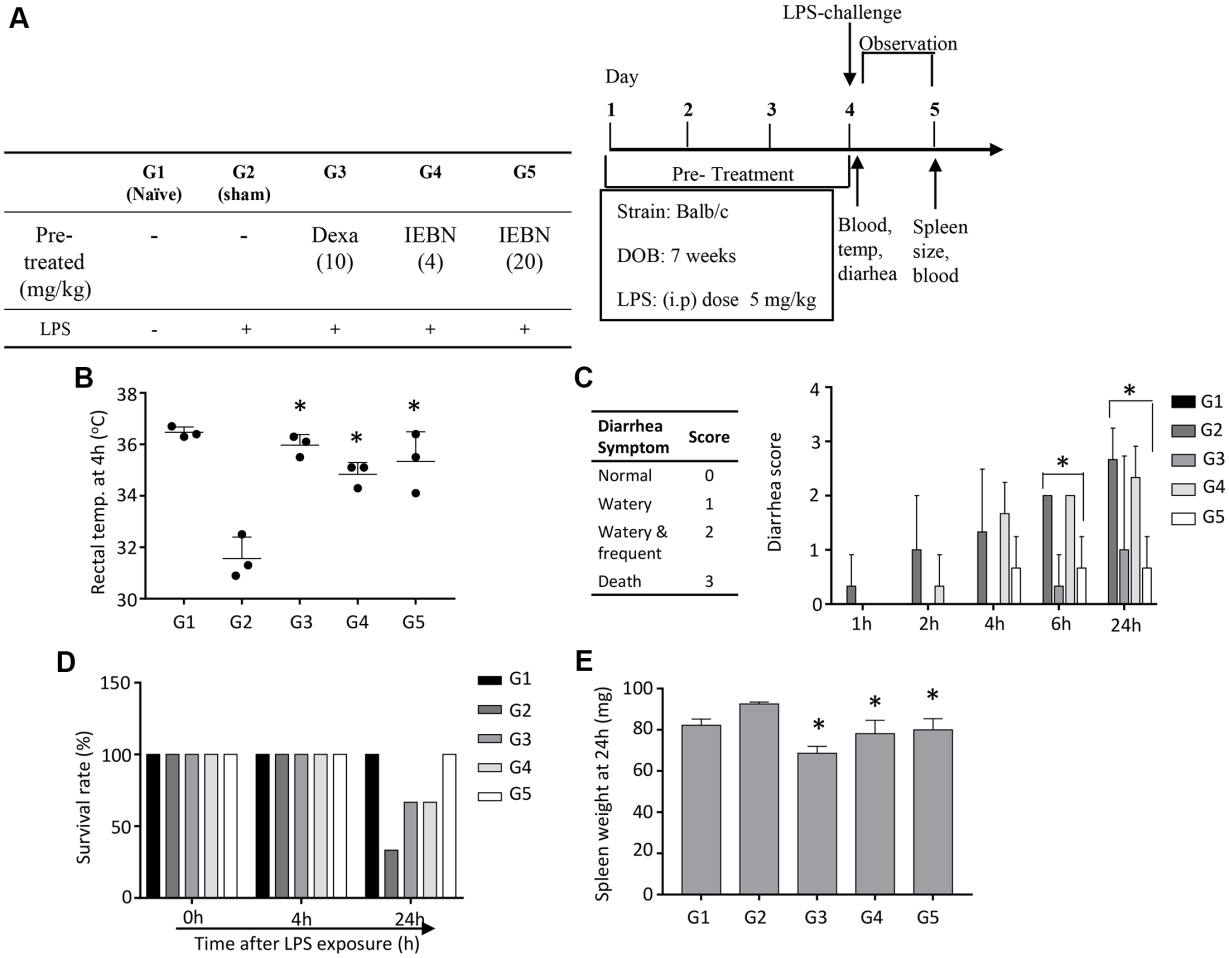
Effects of isoembigenin on the clinical symptoms by LPS-activated macrophages in sepsis systemic mouse model. Mice were pretreated for 4 consecutive days of compounds, and then were challenged by LPS (5 mg/kg) 1 h after last treatment. The clinical symptoms were observed for 24 h. (**A**) The experimental scheme was shown. (**B**) The change in rectal temperature was measured 4 h after LPS-induced inflammation. (**C**) Diarrhea occurrence were monitored during 24 h. (**D**) Survival rate and (**E**) spleen inflammation were monitored and measured 1 day after inflammation onset. The data are presented as the means ± SD of three mice per group. Statistical significance was presented by one-way ANOVA followed Bonferroni post-hoc test and represented as follow: *p* < 0.05(*) vs. sham group.

Macrophages are key myeloid cells in the immune network during septic shock. Therefore, we also examined the effects of IEGN on macrophage activation *in vivo*. IEGN was administered orally for 4 days before the LPS challenge. When the mice were challenged with LPS (i.p.), several clinical symptoms of sepsis, such as diarrhea, decreased body temperature, and mortality, were clearly observed from 2 to 24 h after the LPS challenge. Pre-treatment with IEGN resulted in significant improvements in sepsis symptoms in a dose-dependent manner, with their body temperatures remaining nearly normal (Fig. 3B). The severity of diarrhea was significantly reduced compared to sham mice. Consistently, stools of IEGN-pretreated mice appeared less watery and more normally shaped 24 h after LPS challenge (Fig. 3C). When a high concentration of LPS was administered, the mice entered endotoxin shock, leading to death. The results in Fig. 3D clearly show that 70% of sham mice died within 24 h after LPS injection, whereas only 30% of mice in the low-dose IEGN group (4 mg/kg) and the dexamethasone group died. In contrast, there was no mortality in the mice pre-treated with a high dose of IEGN (20 mg/kg) (Fig. 3D). Interestingly, the efficacy of IEGN (20 mg/kg) in ameliorating sepsis in the murine model appeared to be better than that of IEGN (4 mg/kg).

The spleen is an important secondary organ of the immune system, where immune cells are trained and matured to combat diseases. Therefore, we next investigated the response of IEGN to the LPS inflammatory challenge model. The LPS challenge caused a time-dependent splenomegaly. In comparison to controls, IEGN pre-treatment protected against splenic inflammation. In contrast, dexamethasone, a potent immunosuppressive agent, also alleviated the symptoms of sepsis (Fig. 3D) but resulted in reduced splenic weight, reflecting its well-known metabolic and immunologic side effects (Fig. 3E).

### Inhibition of Pro-Inflammatory Cytokines by Isoembigenin in Mice

**Fig. 4:**
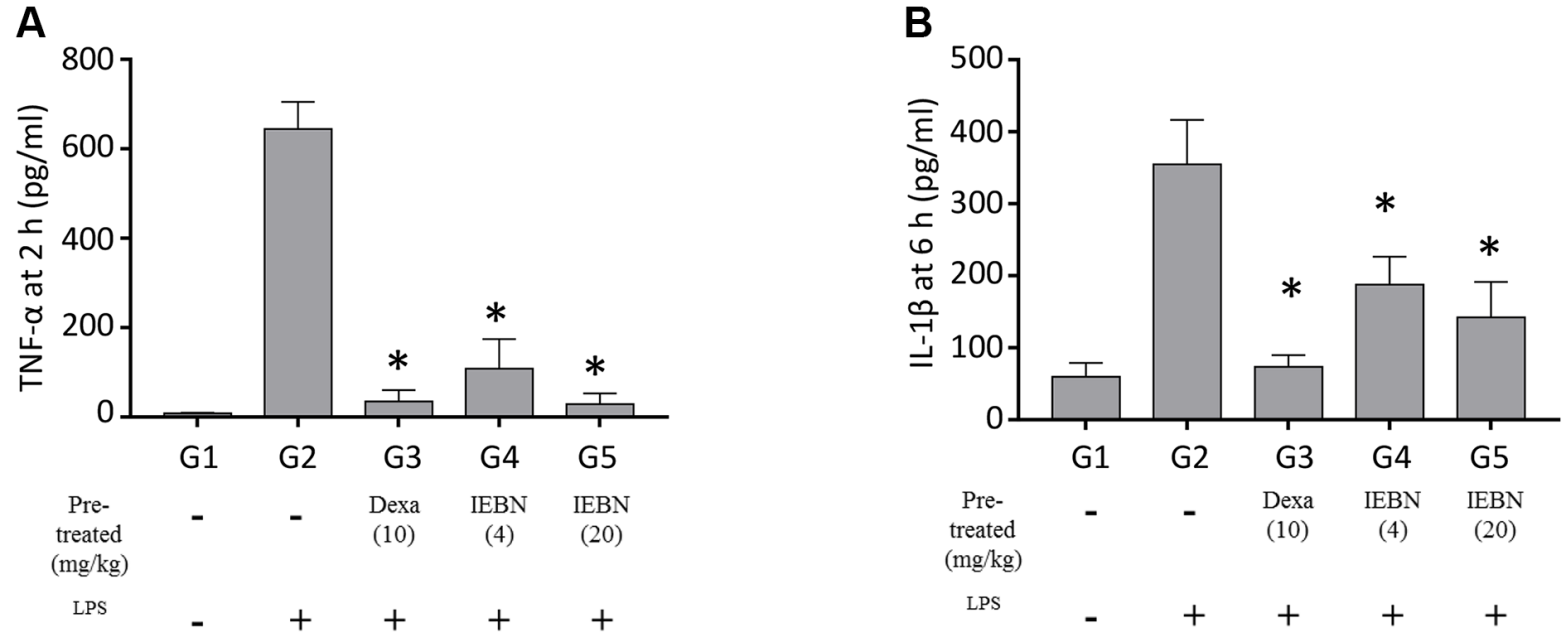
Effects of isoembigenin on the pro-inflammatory cytokine productions by LPS-activated macrophages in murine model. Mice were pre-treated with compounds for 4 consecutive days with compounds, and then were challenged by LPS (5 mg/kg) at 1 h after last treatment. Mice were harvested blood 2 h and 6 h after LPS-induced inflammation for test ELISA. The level of (**A**) TNF-α expression at 2 h and (**B**) IL-1β expression at 6 h in serum were measured by ELISA. The data are presented as the means ± SD of three mice per group. Statistical significance was presented by one-way ANOVA followed Bonferroni post-hoc test and represented as follow: *p* < 0.05 (*) vs. sham group.

LPS administration induced an elevation in TNF-α levels after just 2 h. When the mice were pre-treated with either IEGN or dexamethasone before the LPS challenge (i.p.) to induce sepsis, the serum levels of TNF-α were markedly diminished compared to those in the control group in a concentration-dependent manner (Fig. 4A).

Similar to TNF-α, IL-1β is also a critical cytokine in the inflammatory response. However, LPS stimulation resulted in elevated serum levels of IL-1β at a later time point. As expected, the sham mice produced noticeably the highest levels of IL-1β compared to the other groups. Meanwhile, the pre-treatment groups, including both IEGN concentrations and dexamethasone, produced lower levels of IL-1β compared to the sham group, indicating that these pre-treatments have attenuated macrophage activation in response to septic shock (Fig. 4B).

### Molecular Docking Simulations in Drug Discovery

Virtual screening through molecular docking has become a cornerstone of structure-based drug discovery since its inception in the early 1980s. This computational approach allows researchers to predict and visualize interactions between small molecules and target proteins at an atomic level, providing crucial insights into biochemical processes. In our study, molecular docking simulations were conducted to validate the binding affinity of ligands to receptors, guided by *in vitro* experimental data. Given the critical role of iNOS and COX-2 enzymes in inflammation—specifically in the production of mediators like nitric oxide (NO) and prostaglandins (PGs)—these enzymes are key targets for therapeutic strategies against inflammatory diseases. Understanding how these enzymes are regulated at the molecular level is essential for designing effective treatments that modulate inflammation and alleviate related pathological conditions.

**Fig. 5:**
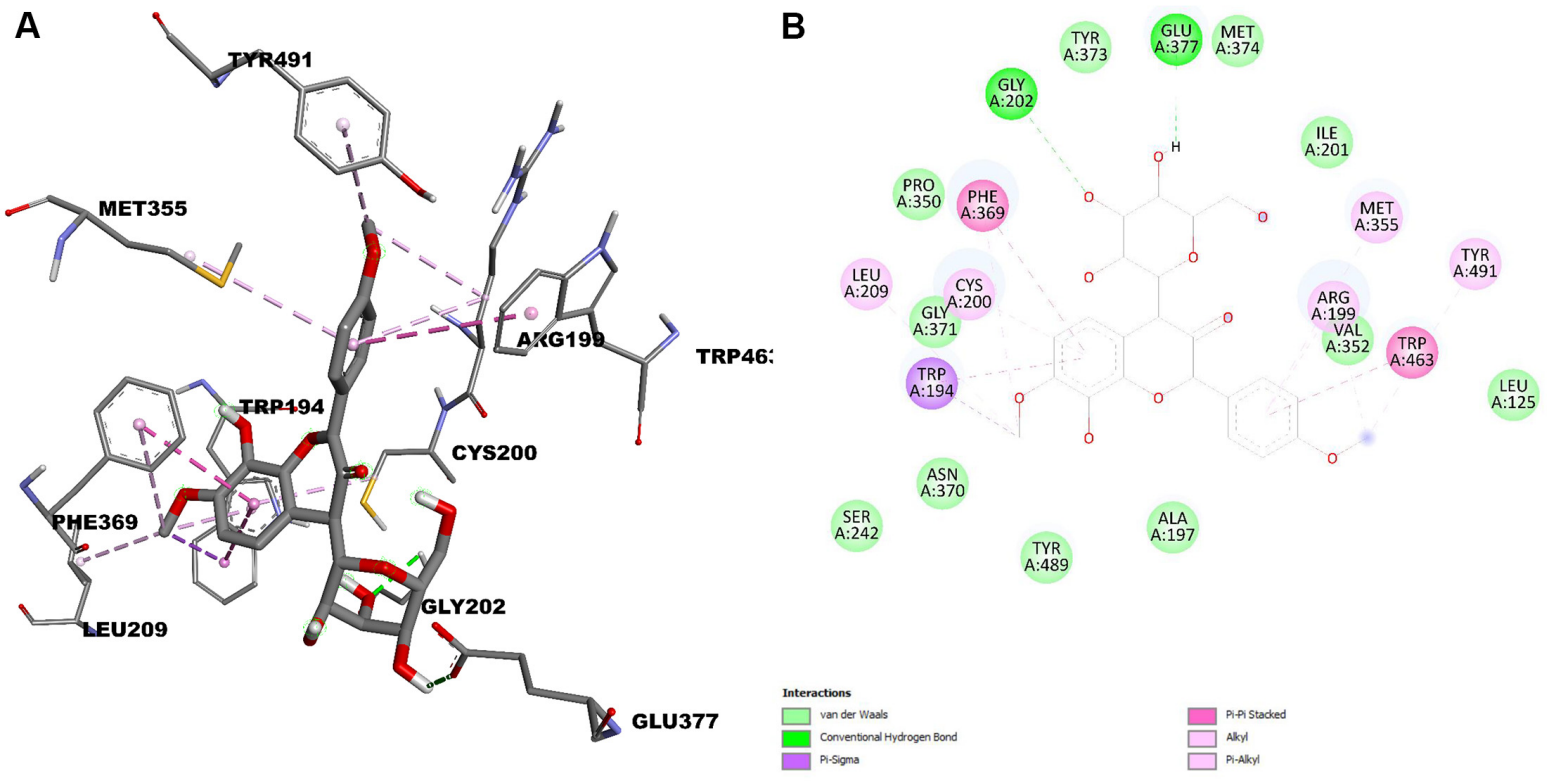
3D molecular docking models for iNOS inhibition (A), and 2D interaction diagrams of isoembigenin’s inhibition of iNOS (B).

**Fig. 6:**
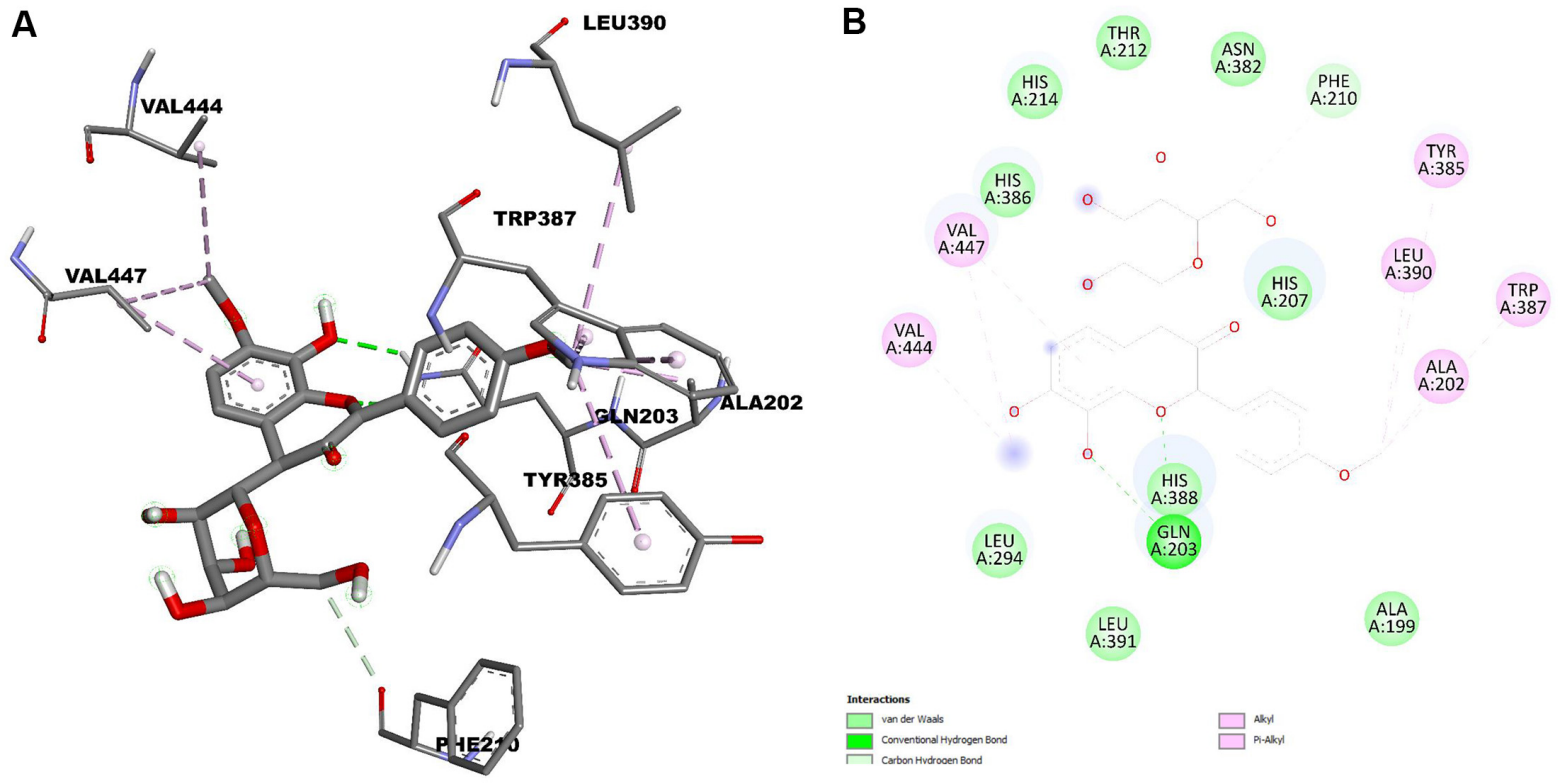
3D molecular docking models for COX-2 inhibition and 2D interaction diagrams of isoembigenin’s inhibition of COX-2.

The crystal structures of iNOS (PDB ID: 4NOS) and COX-2 (PDB ID: 5F1A) are available in the Protein Data Bank, revealing the active site, key interactions, and the molecular mechanism of these inhibitors against inflammation. The docking results are presented in Figs. 5 and 6.

Isoembigenin was then docked into the active sites of the iNOS and COX-2 proteins using the same established procedure. According to our docking results, isoembigenin can bind to the active sites of iNOS and COX-2 proteins, with docking scores ranging from −8.610 to −9.156 kcal/mol. This indicates a potentially strong binding affinity between the ligands and the target inflammatory proteins (Tables 1 and 2). In other words, it suggests that the ligand is predicted to bind tightly to the protein, forming stable interactions at the binding site. Notably, GLY202, GLU377, and TRP194 play an important role in inducing and stabilizing the active conformations of the iNOS protein (Fig. 5). For the COX-2 protein, the aglycone of isoembigenin established hydrogen bond interactions with GLN203 and PHE210 from the active site (Fig. 4). However, the molecular docking simulation provides a hypothetical insight into the potential interaction between IEGN and iNOS and COX-2. While these results are predictive and do not constitute experimental validation, they offer a valuable starting point for guiding future *in vitro* and *in vivo* studies aimed at confirming the role of IEGN in modulating these targets. These results suggest that isoembigenin may be useful for treating inflammatory diseases.

## Discussion

In recent decades, an increasing number of flavone C-glycosides have been isolated from rare medicinal plants, displaying a wide range of pharmacological activities, such as antioxidant, anti-tumor, and anti-inflammatory effects [20]. Apigenin is a natural flavone compound found in foods like parsley and chamomile, known for its strong anti-inflammatory properties. It works by inhibiting key inflammatory pathways such as RAGE (the receptor for advanced glycation end products), which stimulate NF-κB, thereby reducing the production of pro-inflammatory cytokines (like TNF-α, IL-1β, IL-6) and enzymes (COX-2, iNOS) [21, 22]. Other studies have indicated that apigenin has effects in cancer models [23, 24]. Isoembigenin shares a flavone backbone similar to apigenin and vitexin but differs in the positions of hydroxyl and methoxy groups; however, the scientific literature detailing its anti-inflammatory effects is limited. A previous study demonstrated that IEGN has an anti-inflammatory effect in BMDC: Bone marrow- derived dendritic cells-induced dendritic cells [12, 25]. Therefore, it is not surprising that IEGN dramatically inhibited the TNF-α levels induced by LPS stimulation in murine macrophage (RAW 264.7) cells. However, there is a lack of *in vivo* data on the biological benefits of IEGN. It is necessary to further investigate how IEGN prevents and manages the inflammatory response.

A novel potential drug candidate must meet criteria that include evaluating efficacy and safety in animal trials. In the first criterion, IEGN demonstrated strong inhibition of the inflammatory response. Macrophage activation induced by endotoxin triggers produces high levels of pro-inflammatory cytokines, such as TNF-α, IL-1β, and IL-6 [21, 26, 27]. Mice exposed to a high concentration of LPS may provoke severe systemic infection and often cause lethal endotoxin shock [28]. The IEGN pre-treatment groups attenuated cytokine production (TNF-α and IL-1β) in serum, leading to an improved survival rate in the mouse model, with no deaths recorded in the higher IEGN pre-treatment group (20 mg/kg). This result is consistent with the dosage reported in traditional medicine literature. According to traditional Vietnamese medicine, the seeds and roots of *P. aduncum* have long been used as remedies for diarrhea, stomach pain, asthma, cough, and tooth decay [29]. To bridge traditional knowledge with experimental research, we selected a dosage (20 mg/kg) corresponding to the amount traditionally used for the roots, thereby providing a scientifically relevant basis for evaluating the pharmacological effects of the plant *in vivo*.

Moreover, a high concentration of LPS was administered to the mice, resulting in systemic inflammation and leading to multi-organ damage, affecting the spleen, liver, and lungs. The spleen not only produces cytokines for host defense but also clears senescent erythrocytes and maintains a blood reserve during the anti-infection process [30]. Most studies have screened for active compounds to prevent the time-dependent splenomegaly caused by LPS challenge in mice. However, it often requires a long-term approach to treat inflammation, which can lead to other side effects. Dexamethasone is a well-known compound for treating chronic inflammatory diseases, but it induces spleen atrophy via the apoptosis of splenocytes [31, 32]. The IEGN pre-treatment groups did not affect splenic weight or splenic volume. Moreover, although the docking results suggest potential binding of IEGN to iNOS and COX-2, experimental validation is required to confirm these interactions and their biological significance [33]. These computational predictions serve as a useful framework for designing targeted experiments in future studies to better understand the anti-inflammatory mechanisms of IEGN.

In summary, the results of our study elucidate that IEGN is a potent inhibitor of macrophage activation, reducing cytokine protein production in both culture supernatants and serum. In the sepsis mouse model, IEGN demonstrated not only a significant improvement in clinical symptoms but also safety for immune organs. Therefore, it is suggested that IEGN should be considered as a potential therapeutic agent for treating inflammatory-associated diseases.

## Figures and Tables

**Table 1:** Binding affinity and binding interactions of isoembigenin and 4NOS.

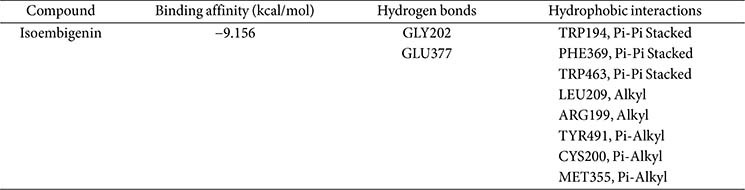

**Table 2:** Binding affinity and binding interactions of isoembigenin and 5F1A.